# What affords being creative? Opportunities for novelty in light of perception, embodied activity, and imaginative skill

**DOI:** 10.1177/10597123231179488

**Published:** 2023-06-16

**Authors:** Michael Kimmel, Camilla Groth

**Affiliations:** 1 27258 University of Vienna, Cognitive Science Hub, Austria; 2 Department of Visual and Performing Arts Education, University of South-Eastern Norway, Notodden, Norway

**Keywords:** creativity, affordances, imagination, crafts, dance

## Abstract

An affordance perspective highlights how resourceful the ecology is for creative actions of all sorts; it captures how creativity is grounded in materiality. In contrast to “canonical affordances” (i.e., “ready-to-hand,” mundane instances), creative affordances point to unconventional or surprising action opportunities that are nonetheless valued. Our initial aim is to discuss how to frame the affordance concept to make it attractive for the study of creativity. We propose a dialectic position that reconciles aspects of the realism of ecological psychology with the constructivist view more typical of creativity scholars. We stress that novel options frequently depend on constructive actions; novelty cannot always simply be “found” or just waits to be used. Many creative opportunities only emerge from how person actively engages with the ecology. Our second aim is to explore specific ways that creativity is mediated through affordances, based on illustrations from crafts and dance. These suggest that affordances span various timescales and mediate in multiple ways, from noticing existing potentials, via active affordance shaping, to background activities that indirectly invite or enable novelty. In conclusion we discuss how a person’s creative “vision,” imagination and combinatoric ability, all fundamental creativity mechanisms, relate to affordances and how fruitful creative directions may be perceptually hinted at.

## Introduction

Creativity scholarship is beginning to respond to the long-time neglect of the fact that creativity not “just happens in the head” and to realize that embodied interactions with the ecology provide creative resources. Aligning with this paradigm shift, our aim is to discuss how affordance theory can support this ongoing re-orientation. Promoting cross-talk between affordances theorists and creativity researchers, however, is less straightforward a task than some recent authors make it out to be. It requires a serious discussion of partly diverging epistemological viewpoints, the locus of causality in creative process dynamics, as well as the scope of perceptual mechanisms relative to action and the imagination. In this contribution we aim to tackle these issues while trying to clarify whether affordances themselves or extended practices are what we typically consider “creative.” We will use forms of interactive and physically realized creative expertise to illustrate our conceptual analysis, the crafting of a vase (creativity of an outcome-oriented kind) and a dance improvisation (creativity of a performance-oriented kind).

### Where is the ecology in creativity theory?

The puzzle of creativity is how novel things enter the world (Kaufman & Glăveanu, 2019; Kaufman & Sternberg, 2019). Creativity forms a spectrum from common everyday forms known as small C creativity to big C creativity (Kaufman & Beghetto, 2009). The big C extreme is radical innovation that creates things unthinkable “before the act” (cf. Boden, 1990), but a great many other things are credited with creativity. Creativity has outcome-oriented forms such as invention, painting and composition as well as ephemeral realizations as in dance or music improvisation (Kozbelt et al. 2010). Some creativity produces historical “firsts”, but usually psychological novelty suffices, referred to as H-creativity and P-creativity, respectively, by Boden (1990). Underlying this multiplicity, there is consensus that for speaking of creativity something needs to be, both, novel and valued in some respect such as functionality or aesthetics (Sternberg & Lubart, 1996). Less consensus, however, can be claimed regarding mechanisms that underlie creativity.

Classically, creativity is held to be “psychological.” Traditional theories seek to understand it as a mental function related to changes occurring in the mind based on mechanisms such as problem solving, analogy building, recombination, divergent thinking, or intuition (Sternberg & Lubart, 1998). Virtually all such *mentalist* theories sidetrack the ecology and what interacting with it does for creativity. They also relegate senses to delivery systems and action systems as actuators of a central processor, that is, the embodied “periphery” is not essential to explaining creativity, nor are interactions with the ecology.

In recent times, a major impetus for ecologically minded, interactionist alternatives comes from performers, artists, and craftspeople who emphasize how creativity benefits from “conversations” with materials, places, things, or other people. In crafts, for example, this is discernible in how the texture of wood guides how a basket maker forms their material in the act itself (Ingold, 2013, Ingold 2010); or a felt maker follows the natural entanglements of fibers (Aktaş, 2019). Form is not necessarily imposed in advanced but grows through continuous engagement, for example, as a sculptor releases the form from the marble, as Michelangelo famously said (Ingold, 2014). “The craftsperson develops a deep understanding of the vitality of a specific material” (Mäkelä, 2016, p. 3) and there is a dialogic negotiation between the maker and the material (Brinck & Reddy, 2020).

Mentalism seems theoretically limited as well. A fundamental shortcoming lies in being oblivious to process and just investigating preconditions relative to outcomes. Only once we go beyond this (and the often associated myth of the original genius and the “heureka” moment) and recognize the required preparation and hard work over time instead can we also begin to see interactive features where creativity evolves through the interplay of “enterprises,” that is, arrays of activities, with concepts (Gruber, 1989).

Another widespread oversight is how integrally embodied action and perception operate with reasoning, as creators recursively move back and forth between “external” exploration and “internal” idea generation or specification. They employ situated “here-and-now” resources (cf. Kirsh, 2009; Robbins & Aydede, 2009). Material manipulation or exploration are often just as crucial as “thinking” and are seen as a form of thinking in itself by practitioners (Mäkelä, 2016; Groth, 2017; Brinck & Reddy, 2020). Although some psychological views broadly acknowledge the generative power of action, functions such as exploration are largely characterized as mental or their locus remains unspecified.^1^

Creativity also cannot be reduced to a solitary practice. Embedding in histories and cultural contexts matters, as do collaborative aspects, for example, when Sawyer (2006) emphasizes that many insights can be traced back to previous collaborations, build on previous contributor’s ideas, or insights are made in conversations with others. Glaveanu (2014b) proposes speaking of a new “we” paradigm of creativity research, which emphasizes the importance of social variables as well as, possibly, the role of real-time collaboration.

The mentalist problematic culminates in the strong attachment to methodological individualism. Traditional scholarship disregards causal effects emerging as creators engage with their ecology. effects which are an outcome of the interaction dynamic itself. According to Valée-Tourangeau (2014, p. 27) “thinking and problem solving outside the laboratory involve interacting with external resources [....].” We must therefore investigate *relational* activity, the “transactional agent-environment coupling” (ibid, p. 40).

### Post-cognitivist theory to the rescue

A number of scholars are trying to remedy these blind spots. Early exceptions that highlight physical exploration and interactivity are Schön (1992) study of design processes, and Bamberger and Schön (1983) related notion of “knowledge-in-action”. Approximately a decade later Sawyer (2003) pioneered the study of *collaborative emergence* processes that underlie group creativity in improvisation theater, jazz, and conversations. The term *distributed creativity* is proposed by Sawyer and DeZutter (2009), building on the broader concept of distributed cognition proposed by Hutchins (1995, 2014). This resonates ideas about so-called extended cognition, where features of the ecology can serve as “transparent equipment” (Clark, 2008).

In the study of material creativity, Glăveanu (2014b, 2014a) presents an influential model of distributed creativity within a cultural psychology framework, in which he integrates actors, audiences, artifacts, actions and affordances as part of creative systems. Malafouris (2014, p. 145f)—in his equally influential theory of material engagement—states that “the making of the creative idea is inseparably […] mental and physical” and the creator’s conceptual space is “in moment-to-moment improvisational thinking inside the world”. In colorful, yet also perhaps discussion-worthy metaphors, Malafouris speaks of creative “thinging” (2014) and Ingold of “creative undergoing” (2014).

“4E” (embodied, enactive, embedded, and extended) cognitive science has made its own bid to model creativity, defining it as a function of embodied coupling dynamics and stressing resources that arise from engaging with objects, spaces, and people. Malinin (2019) proposes a definition of creativity that describes it “as situated practice, emerging through person-environment interactions (material/technological as well as socio-cultural).” Work from the enactive branch of “4E” cognition (Davis et al., 2015; Malinin, 2016) is worth mentioning here. It builds on Varela et al. (1991), who famously claimed that perception and cognition depend upon interaction with the world. In an embodied coupling perspective, creative systems operate in “continually flowing and dynamic interaction with an environment rather than discrete actions and goal-oriented planning” (Davis et al., 2015, p. 119).

Similar claims emerge from research on interactivity-based cognition, showing that acting in one’s ecology produces more rapid, robust, or parsimonious cognition than internal thought alone, for example, by using space as an external memory (Kirsh, 1995; 2014; Kirsh & Maglio, 1994). Creative problem solving (Steffensen, 2013a; Steffensen et al., 2016; Trasmundi & Steffensen, 2016) is facilitated through changes of layout, moving around, physical manipulation, active exploring, and stimulating feedback. In addition, the finding of creative pathways benefits from external or task constraints that narrow down the possibility space (Hristovski et al., 2011; Torrentset al., 2021). As the layout of systemic parameters changes processes self-organize in potentially creative ways, as demonstrated for sports and dance (Chow et al., 2011; Orth et al., 2017) as well as jazz duets (Walton et al., 2015; Walton et al., 2018). Such dynamic systems methods frame creative process as self-organizing systemic coupling dynamics (Hristovski et al., 2010; 2011; Torrents et al., 2021a; Torrents et al., 2021b). In addition, to reconstruct the dynamic process bottom-up micro-genetic methods have been employed, both in phenomenological studies (Kimmel & Hristova, 2021) of collaborative co-creation and in observationally oriented micro-genetic studies (Ross & Vallée-Tourangeau, 2021a; Steffensen et al., 2016). These perspectives share in common the assumptions that creativity transcends the cranium, that the proper unit of analysis is the agent-ecology system in its entirety, and that interactivity has causal power.

## Creative affordances

What then can the role of affordance be in this scholarly re-orientation and how can the concept be harnessed to the specifics of the creativity debate?

### Definitions and applications

A pillar of James Gibson’s ecological psychology (e.g., Gibson, 1979), affordances canonically refer to properties like the sittability of a chair, the graspability of a handle, the drinkability of liquid from a glass. Affordances are specified by ambient information patterns pointing to actionable features and held to exist independently of whether they are perceived by someone (*realism*). To the extent that they *are* perceived and acted on, they mediate perception-action coupling as we move in space, manipulate objects, or interact with others. Affordances lie betwixt and between—they are typically both the results or prior actions and precursors to next ones, as agents move through and shape their ecology (Chemero, 2009; Reed, 1996; Richardson et al., 2008). Perception is for action and—inversely—actions create further opportunities for perception for ecological psychologists affordances inhere in the specific relationality between agent and environment. Since these co-constitute each other, they are also to be taken as a single unit of analysis .^2^

Affordances provide researchers with a concept to explore the guidance found in materials, objects or social spaces and to investigate “creative actionables” supplied by these ecologies. To apply the notion it is, first of all, crucial to emphasize that the ecology is richer in “actionables” than normally lies in focus. Creativity moves into focus affordances that are seldom realized or used, that present alternatives to what is customary, push the norm or technical limits, or that relate to yet inexistent entities. This is possible because agents almost always operate in a broad *landscape of affordances*, the manifold opportunities in a skill domain or ecology, which in each situation leaves a sizeable *field of affordances* to choose from (Bruineberg & Rietveld, 2014; Rietveld & Kiverstein, 2014).

Glăveanu’s (2014a, 2014b) cultural psychology framework claims that affordances for creative actions are not easy or obvious, but push the boundaries of the possible or the socially accepted. Of course, a great many affordances are placed in the zone of what is usually done. Here, everyday intentions, typical affordances, and cultural norms overlap. At the fringes however there are various unperceived affordances, pointing to experiments where many subjects, owing to functional fixation, fail to *perceive* a helpful affordance. Some perceived affordances remain *unexploited* for cultural reasons if they are norm-violating or sanctioned; yet creators may begin to exploit these nonetheless and reshape cultural norms. Other creative affordances are yet to be discovered, or inexistent as long as their carries haven’t been *invented*. Furthermore, creators must frequently draw on partial affordances and assemble compounds from them, that is, “new collections of affordances generated by the combination or transformation of basic (existing) potentials” (Glăveanu’s 2014b, p. 219). Glăveanu’s (2020) more recent *affordance-perspective theory* stresses that recognizing affordances depends on adopting a larger contextual orientation: “Creative action fundamentally depends on the development of perspectives from which new and unusual affordances are revealed […]” (p. 345). Serendipitous finds, for example, do not stand for themselves, but require “a perspective that recognizes the affordance” (p. 346), as in the famous example of the weak glue that led to the development of “Post-Its,” an affordance which had stayed unnoticed for a time before being utilized.

Costall (2015) emphasizes the role of unconventional uses of objects in creativity and points out that “in everyday life, we are usually very effective in co-opting objects in non-standard ways into our ongoing activities, for example, catching a spider under an upturned glass” (p. 51). Similarly, Withagen and van der Kamp (2018) stress the importance of exploratory behavior, and the ability to perceive (and created) unconventional affordances.

The *skilled intentionality* framework developed by Rietveld and colleagues argues for the fundamental richness of resources found in the environment. Yahklef and Rietveld (2020, p. 8) explain the concept of “innovation” as sensitivity to wide existing landscapes of affordances: “[i]*n* situations of innovative action, people ‘join forces’ […] with relevant affordances available in their socio-material practices. Although the environment may be replete with affordances, individuals will normally be solicited by mainly those that are salient […].” Innovation then results from a dialogue between affordances available in a socio-cultural field and the embodied skills shaped by it, a relationship in which disequilibria can trigger discovery as well as attempts to experiment and manipulate so affordances are revealed. Spontaneous, but unconventional responsiveness to the environment is “partly constitutive of innovative action” (p. 10). New skills or skill combinations as well as “imports” from other fields can widen this responsiveness. At the theory plane, the skilled intentionality framework integrates affordances with Wittgenstein’s notion of “form of life,” of which stable socio-cultural practices are an example (van Dijk & Rietveld, 2017), as well as with the idea that responsiveness to affordances improves “grip” on a situation (Rietveld & Brouwers, 2017). The authors ethnographically illustrate this for an architect’s workspace, stressing that architectural design or visual arts can encourage the exploration of new affordances by scaffolding experimentation through so-called “material playgrounds” (also see Kaaronen & Rietveld, 2021). Finally, skilled intentionality authors try to extend the affordance perspective to the imagination (van Dijk & Rietveld, 2018), an attempt we will later discuss.

Baber (2021) presents a Gibson-inspired critique of information processing theory in design thinking, which inter alia left its mark on the reception of affordances by Donald Norman and William Gaver. Baber advocates a radical embodied cognition perspective, and defines affordances in an interesting way: as possible points of stability in a coupled human-artifact-environment system. Creativity can arise when system constraints are selectively loosened so new systemic state (hence, affordance) transitions become available; creative agents opportunistically respond to constraints of the system, but also modify, and probe the limits of the latter (p. 169).

The fact that creative opportunities often owe to continuous interaction is demonstrated by Baber (2015, p. 25) who argues that a goldsmith’s “intent is only loosely defined a priori but crystallizes through the continued interactions between craftworker and object through a process in which the affordances of the object become apparent to, and responded to by, the craftworker.” How interacting with and changing the ecology can generate affordance-disclosing information is supported by Valée-Tourangeau’s work on insight problems (2014, p. 28). He states that “thinking is the product of a fluid and dynamic interaction with external resources that produces a shifting configuration of physical features and action affordances.” A similar emphasis is evident in studies of co-creative improvisation (Kimmel et al., 2018), sports, and dance (Correia et al., 2012; Hristovski et al., 2011; Passos et al., 2012; Vilar et al., 2013).

### Practical and conceptual concerns

While this growing literature ushers in an ecologically minded perspective, several questions await response, including whether affordances may themselves be “creative,” what explanatory scope perceptual responsiveness has, and what other creative functions affordances could fulfill. In addition, to get a dialogue with creativity scholars started a number of basic issues await discussion, from preferred examples, via theoretical definitions to epistemological issues. These are what we turn to next.

Although Gibson doubtlessly intended his theory to be inclusive, the literature presents us with mostly non-creative examples, such as sittability, graspability, reachability, pass-through-ability, or climbability. These are “canonical affordances” (Costall, 2012) of normative and well-defined tasks; they are mundane and, in a Heideggerian vein, “ready-to-hand” (cf. Dotov et al., 2017); they are typically not perceptually ambiguous nor precarious to execute. Unfortunately, all these properties may not apply to creativity contexts. Such accounts often evoke discrete, immediately perceivable givens and quick, routine, actions that follow them, typically executed “one-shot” without preparation, exploring, or problem solving. We hear “sittability” and picture ourselves in a scenario where a chair already stands next to you—and we just sit down. Seldom do we think of scenarios where we have to first search for a chair, unfold it, assemble its pieces through problem solving, repair it, even invent or design it. Nor do we typically picture events in which we must find an alternative means, or would consider using the chair inappropriate. Thus, canonical affordances make for bad exemplars for creativity applications.

Ultimately, however, deeper conceptual issues are at stake. As Costall observes, according to the Gibsonian tradition the users of affordances are “recipients of already established meanings” (2015, p. 51). He also quotes Shotter’s (1983, p. 20) criticism that beings in Gibson’s world “may move about, but they do not act; rather than ‘makers’, they are merely presented as ‘finders’ of what already exists.” The relative neglect of skilled manipulation as well as the fact that new things or behaviors can be actively created limits inquiries to the present day, as we shall discuss later.

In particular, to apply to creativity, we should abstain from depicting affordances as stable or factual. Chemero (2009) remedies this problem by defining affordances as dynamic and responsive to interaction. Dynamicity can imply two things about affordances: One claim is their “quicksilvery” (Fajen et al., 2009, p. 89) nature, especially in contexts where the ecology moves and responds rapidly, for example, in sports. Affordances will rapidly evolve and devolve in terms of how actionable, or perceivable they are. A further reaching second claim is that affordances *causally* manifest through dynamic engagement with the ecology (Kimmel & Rogler, 2018). How a person explores or manipulates the ecology contributes to revealing specific affordances. Accepting just how much specific affordances depend on specific trails of interaction is fundamental for an affordance perspective on creativity. Engaging with the ecology can make new affordance-specifying information salient; generated feedback can reveal new creative opportunities or suggests further exploratory moves (Gaver, 1991).

Another possible incongruity between affordance and creativity research results from Gibson’s emphasis on the wealth and sufficiency of ecological information. This sit somewhat uneasily with creativity contexts in which information scarcity, ambiguity, and brinkmanship may loom large and which require effortful work, gradual development, or compromising one aim for another. If we assume that affordances arise in and through extended interaction this may mitigate this theoretical incongruity somewhat. A person who can recursively engage with the ecology over time is better poised to handle this precariousness, an “open-endedness” and continually coming into being of affordances that is stressed by Costall’s (2015) discussion of creativity.

### Epistemology between realism and constructivism

To understand how affordances can explain creative processes we need to critically reflect on fundamental epistemological issues.

Unquestioningly, a Gibson-inspired realist epistemology can claim particular merits for explaining creativity. Christensen (2002, p. 55 f), one of the very few authors discussing such a general perspective from a creativity angle, explains this as follows: “Realist theories focus heavily on the objective structures and objects, and how they guide and constrain the creative process. Objective structures, rather than subjective processes, are analyzed to explain creativity. Realist theories do not construct out of nothing, but instead try to explain how something came to be something else.”

This notwithstanding, substantial tension remains between Gibson’s framework and the legitimate concerns of creativity scholars. Most creativity researchers are epistemological constructivists and emphasize active creation. What this means for creativity is, again, nicely captured by Christensen (2002): “Realists ‘find’ solutions, whereas constructivists ‘create’ them” (p. 57). What puts the realist position under pressure is that novelty “is, by definition, not out there to be found (if found means simply ‘picked up or bumped into’)” (Christensen, 2002). Creativity scholars would emphasize a fundamental non-determinism of creative practices. (We might add that, even if a realist could legitimately respond that things yet unrealized are “real as possibilities,” this does not much advance the agenda of studying *how* they become real.) Furthermore, there is strong consensus that creativity *may* involve mental generativity mechanisms. Gibson’s radical philosophical externalism offers little room for this fact and, for example, makes it tricky to discuss how elements are creatively re-combined. To speak with Christensen, the danger is that realism can “result in attempts of trying to eliminate the subjective processes and abilities to transform and construct.” (p. 55f). A perspective devoid of mental mechanisms will seem odd even to many creativity scholars who accept the resourcefulness of the ecology and who critique mentalist reduction themselves.

Importantly, realism also limits how we can understand the specific relationship of creativity to affordances. Realist authors tend to emphasize the skillful spotting of affordances, yet creativity is not reducible to search and discovery. Mere affordance “finding” is not enough; abilities to transform and construct existing affordances are equally essential, a point argued more at length below.

It seems that, for genuine rapprochement between creativity research and ecological psychology, we will have to negotiate an middle ground. Although this lies beyond our present scope the dialectical synthesis proposed by Christensen is a good starting point readers may want to consult. He argues that a complete theory of creativity needs to “include both the aspects of creativity focused on by realists (objective structures and products, what, search, usefulness), as well as the aspects focused on by constructivists (subjective generative processes, how, novelty).” (p. 57). Christensen also stresses that the two perspectives explain different aspects of creativity.

Overall, this would suggest seeing creative process as an interactivity-based movement towards the “not-yet-real.” We can thus think of much creative activity as continuously moving along a “proximal zone of development,” to use a Vygotskian (Vygotsky, 1978) analogy , a horizon that expands what is real through processes of active engagement.

## Two case-studies

To clarify how affordances mediate creativity we will now summarize two empirical studies: (a) a crafts context of human-material interaction and (b) a human-human interaction from a dance context (Kimmel & Groth, 2023; Kimmel & Hristova, 2021). For brevity’s sake we will bracket out methodological details and detailed process descriptions. Taken together, the studies indicate that task context greatly influences the operational loci and range of affordances, as well as emergent causalities.

### Material interaction: A crafts example

In crafts (“making”) contexts, a material such as metal, paper, wood, textile, clay, etc. is transformed into a functional and/or aesthetic object by exploiting properties such as malleability and by using tools. Expert practitioners possess substantial sensorimotor skills and experiential knowledge of materials. As they work, they conform to an overall process logic, a chain of operations, that is, a series of actions of a set order, duration, and relative timing (Leroi-Gourhan, 1993; Nørgaard, 2018). Although practitioners need to conform to these procedural and technical constraints, many respond to material emergence to finesse the object or find directions, rather than just following a specified pre-design. In other words, there is leeway for creative exploration *during* the process. The evolving feel, visual appearance, sound, and smell of materials and tools orients the finer aesthetic decisions (as well as, of course, allowing practitioners to ensure a technically sound process and structurally sound outcome). Hence, interacting with the material presents practitioners with a stream of affordances, many of which mediate micro-techniques and some of which “route” the main aesthetic decisions of the practitioner. The affordances emerge in a path-dependent fashion, that is, affordances acted on earlier determine material possibilities at subsequent stages.

Our example is from a ceramics context, and displays a clay throwing process of 30 minutes. The ceramicist and researcher (Groth) seeks to make a vase and give it an aesthetically pleasing shape by exploring options “on the go.” Once she has decided about the general type of object, the process begins with preparing the material, workspace, and tools. To ensure that the material will be suitable for the task the ceramicist first rids the clay of air bubbles and excess moisture through kneading it on a plaster board while monitoring the changes. The specific qualities of the material greatly influence this preparation as well, since a particularly translucent, but difficult to handle porcelain clay is used. When the material has the right level of moisture and tactile feel the next stage can begin.

Now, a clay ball is formed and centered on the rotating potter’s wheel. Precision is crucial here. Then, a hole is made in the rotating clay so that a bottom and first indications of a wall appear. Next, walls are raised up to a cylinder of about 20 cm (this is a generic procedure for vases, whereas plates or bowls would require different steps). In doing so, the ceramicist monitors a constant stream of transient affordances which indicate if the object is still centered enough, if the walls are perpendicular, regular and thick enough to be stable, yet not too thick, and if the amount of moisture is well regulated. Whenever something deviates too much in-process corrections are performed by changing hand position, adding moisture, and regulating the speed of the wheel. A large amount of experience and technical skill underlies this mostly functional process, and much could be added about it, but we’d rather fast-forward to the more creative part. This begins once the cylinder is tall enough and has thin, but stable upright walls. The combination of visual and tactile impressions, we might say, produces a main routing point, the “green lights” for the stage of creative work.

**Figure 1.**
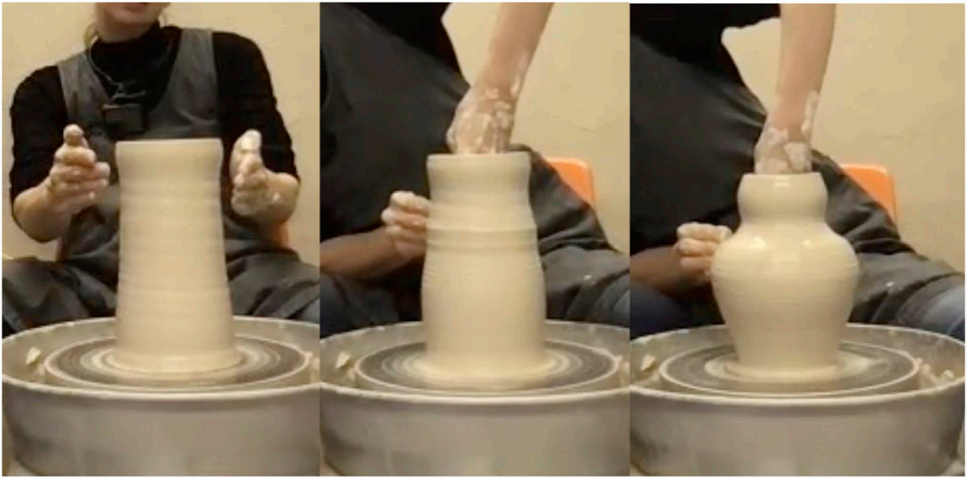
The ceramicist finds serendipitous inspiration as a result of a mundane technical correction. Screenshot of a video recording by the authors.

At this juncture, the ceramicist’s decisions are beginning to be influenced by a phenomenon known as *serendipity* (Ross & Vallée-Tourangeau, 2021b). The first time (of three instances) this occurs as she makes a small technical correction on the cylinder by removing excess clay from the rim. A slight indentation emerges below the rim as an unintended by-product (Figure 1). The ceramicist might have easily corrected this, but instead she decides to go with the flow and accept this feature as aesthetically interesting. Almost immediately her decision to do so imparts to her an inspiration to give the vase as a whole a curved calabash like silhouette. She reports imagining features in the lower portions of the vase that might fit nicely with the curvature emerging below the rim. Therefore, the decision to accept serendipity momentarily triggers a (sketchy) imagination of the vase’s silhouette as it might look towards the end and what a coherent complementation of the accepted feature might be. We might say that an aesthetic reasoning processes seems *afforded by implication* after the decision to accept the serendipitous effect.

Note that, although two further moments of serendipity shape the subsequent process, material emergence is actively rejected at several points. In the final few minutes, the ceramicist rejects two chance events as non-conforming to the creative outlook that has emerged. In addition, there is a constant undercurrent of immediate technical counteractions to material tendencies that would make the vase’s walls brittle, instable, asymmetric, etc.

In the last minute of the process, the ceramicist senses the clay is getting “tired” from being manipulated too long; material feedback warns her of the clay collapsing. In an act of brinkmanship she decides to smooth out the surface with a tool anyway, and embraces a certain risk in doing so. She tries to actively preserve the established shape. As feared, the vase’s walls slightly begin to sag at this point, and she stops. The outcome subjectively feels interesting as far as aesthetics are concerned, and somewhat, although not extremely creative.

To recap, we have described how affordances orient the process in both its technical and its creative dimensions (for details, see Kimmel & Groth, 2023). Transient micro-affordances guide constant technical finessing and corrections, but a few among these also “spill over” into larger creative decisions. At a middle timescale, affordances guide the required *chain of operations*. Material feedback informs where the process stands and possible next steps, for example, if the clay ball is centered enough to begin or the basic cylinder is straight enough to move on. At the global timescale affordances inform about the general state of the material, notably whether the clay has just enough moisture or is getting too “tired.” These affordances need to be actively preserved to keep the clay from collapsing.

### Multiple modalities, aesthetics, and the imagination

All in all, the making process reflects what scholars sometimes describe as a “conversational” negotiation between makers and materials (Brinck & Reddy, 2020), a process in which affordances provide guidance. This underwrites the importance of emergent affordances as a central mechanism of non-preplanned action. In our case, the affordances were deliberately accorded the power to mediate creative decisions “en route.” And the practitioner showed the corresponding ability to source many choices from the interaction with the material itself (albeit against a rich backdrop of knowledge of process constraints and technique). This dynamic emergence notwithstanding, the practitioner also handled actions interdependently; any selected affordance had to make sense with respect to earlier and prospective ones.

What then does the case-sketch reveal about the specific relationship of affordances and creativity? Crafts creativity necessarily operates against a backdrop of standard technical processes, which we cannot ignore. A wide range of affordance orientations are instrumental, from local opportunities to monitoring the task progression and the material’s overall “workability.” Our case-sketch also showed that, in this “material conversation,” the practitioner enjoyed considerable creative autonomy. A typical practitioner will constrain and guide the interplay with material affordances, rather that only responding to them, especially when a particular aesthetic/stylistic strategy or personal inspiration is involved.

Clearly, affordances contribute to creativity in more than one way, from serendipitous “finds” to aesthetic features developed with great creative autonomy and a great deal of active shaping effort. Serendipitous “bumping into” or even deliberate perceptual search for affordances is not always the main mechanism. Much of creativity requires developing material opportunities, led by the imagination. What is more, even serendipitous moments do not really appear to be examples of passive affordance “finding.” Take again the moment that give the process its initial direction: An aesthetic affordance was perceived in the vase’s neck. However, this was immediately and purposefully accentuated to fully realize the serendipitous potential. Also, the perceived chance feature was immediately complemented by (in fact, evaluated in terms of) the aesthetic imagination. We shall return to the complex relationship of affordances to the imagination later, but would briefly like to explain why the latter is fundamental here.

Creative effects arise only from coordinated actions on multiple affordance layers of the clay object. How creative the outcome is deemed to hinge on how the whole vase comes together. (Creativity may depend largely on “tasteful” relative proportions and aesthetic coherence or functional cleverness and only to a small degree on “flashy” signature details). Thus, particular facets of the vase, which may be focus of work at a given moment, are aesthetically appealing only with respect to past or future actions on other features. What technique feels “best afforded” right now is typically evaluated in the light of existing, expected, or indeed imagined further affordances. The practitioner clearly expressed using the imagination (cf. Koukouti & Malafouris, 2020) to ensure that present choices cohere with previous and prospective ones.

Finally, crafting a useable object is a very constrained process. In no way is creativity the practitioner’s sole concern. For an affordance to be chosen it must typically conform to procedural and technical criteria, as well as ensure product functionality and aesthetics. These parallel constraints may sometimes also exhibit trade-offs. A practitioner may trade high functionality for middling novelty or compromise in other ways, for example, when something seems aesthetically interesting in itself, yet clashes with the time budget or mismatches the chosen material. In particular, procedural constraints of correct stage order or maximal duration of actions, have a highly selective influence (Kimmel & Groth, 2023).

### Social interaction: A dance example

Our second vignette focuses on a interpersonal interaction with a similar “conversational” quality to it, as well as uniqueness in terms of creativity. Our example stems from Contact Improvisation (CI), a partner dance which aims at exploration of kinesthesia and weight sharing with many possible, even unique forms and no strict movement grammar as one would see in dances like salsa or tango. CI, qua improvisational domain, is a context of *ephemeral* creativity. Each moment stands for itself; there is no ultimate aim beyond the joint creative exploration itself. Accordingly, each moment is momentarily negotiated between the dancers. Even though lines of action or theme explorations may extend over several seconds or even minutes each moment has its own creative (and experiential) value. Thus, each micro-interaction produces the affordances for the next without any requirement for extended task arcs or the cumulative effect build-up we saw in the crafts context.

The dance emerges in real time. Rapid responses and continuity are essential, and creative decisions are often just “good enough,” not peak creativity (such as one might see in a choreographer). Improvisers must keep the interaction going and respond to every new situation without hesitation, while keeping the sensorimotor system open for surprise at any moment (although creatively relaxed moments as well as “ready-made” mini-sequences occur, notably when attention flags). Myriad things are possible, which makes affordances less predictable than in crafts. The potential for engagement-based emergence is enormous, as we shall see.

Dancing together produces constantly shifting synergistic configurations between the two bodies. This means that affordances of individuals are dynamically connected and interdependent. Most of the time dancers touch, let their forces impact one another, share weight, or even create joint “architectures”, similar to martial arts (Kimmel & Rogler, 2018). Although individual actions are at times loosely physically connected (so-called out-of-contact situations), even then actions are subject to constraints of togetherness. Importantly, the dancers cannot act merely on the basis of what is afforded to them individually but must consider what is afforded in terms of relative distance, body geometry, relative timing, or weight and impulse transmission. This interdependency means that individuals cannot just be creative independently, but seek a creative inter-body synergy (Kimmel & Schneider, 2022). Also, they must at times curb individual options for safety reasons, for example, when leaning on the partner, in a supported handstand, or when creating a lift together. It may be said that affordances of individuals evolve within a dyadic system that links the partners in reciprocal agencies. This dynamic relationship between partners not only faces the individual with new affordances at each moment; the presence of an autonomously moving, yet interconnected partner frequently enhances, blocks, modulates, or destroy a person’s affordances in the fraction of second.

**Figure 2.**
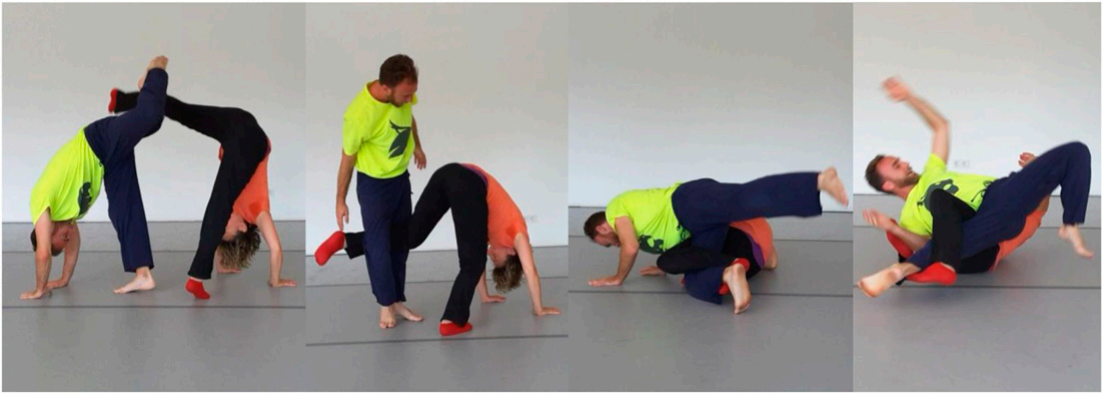
A short Contact Improvisation sequence. From a video recording screenshot made by the authors.

For illustration we now discuss selected moments of a short dance sequence reported in (Kimmel & Hristova, 2021), shown in Figure 2. It begins in an out-of-contact situation in which the dancers are only visually connected. The female dancer autonomously decides to do a high kick. It is simply afforded by the body position and the free surrounding space. The male dancer reacts with a similar high-kick right into the arc of his partner’s raised leg. He reports liking what he sees and feeling inspired. We could speak of an aesthetic complementation of individual affordance, because the two high legs together create an interesting spatial trajectory with a rhythmic syncopation effect.

Next, somewhat unexpectedly for both partners their legs come down in an interlocked position at knee height; the dancers now slightly touch. The male dancer notices that this situation affords increasing the physical connection. By bringing his other knee into a clamp he locks the partner’s leg. This action is almost effortless and serendipitously picks up on how the legs came down. Yet, it is an instance of active accentuation, which shapes affordances. The effect is paradoxical since an impasse for both dancers was created; both struggle to retain their balance. Affordances for free movement are eliminated on purpose. The male dancer reports doing so because this is an interesting problem to boost their creativity. Improvisers like to create challenges for themselves or others and creativity theory has emphasized the value of problem finding (Runco, 1994). The dancers respond to this challenge by carefully negotiating a path to the ground to regain enough support. In doing so, they entangle their knees and legs further, which results in the next emergent constellation.

The female dancer, who wishes to continue the circular movement trajectory of her high-kick, begins to roll sideways. Simultaneously, the male dancer tries to extricate himself from their tangle through straddling in upward direction with his free leg, in order to roll over his partner whose side and back are on the ground. At this moment, both dancers try to create greater degrees of freedom for themselves, to widen their individual affordance fields, but also play in deliberate ways with their state of entanglement, exploring how the partner impacts the possibilities they are now exploring.

Something highly dynamic results: The leg entanglement generates emergent, rapidly changing affordances, at a sub-second time scale. A miniature-scale give-and-take unfolds in which the male dancer’s free leg rises and his body front begins to open, which guides his partner’s pelvis into one direction. She picks up on this affordance and amplifies the micro-effect into a fuller rotation. In doing so, her sideways moving knee begins to impact her partner’s leg. Then, she rotates further and begins to roll away under him. The moving knee now redirects the male dancer’s straddle role mid-way. Then, through her rolling his support is removed. Gravity takes over and he must completely re-orient himself for a sudden drop. Completing his straddle roll that is already under way is no longer afforded. His partner has effectively robbed him of the opportunity, enforcing an emergency response.

Underlying this, a slightly more temporally extended orientation is evident. The dancers both say they are interested in exploring a rotational theme. While creating and responding to short-lived affordances, they also infuse the situation with their creative interests, notably—they emphasize—exploring further possibilities for rotational forms. This orientation has not been explicitly agreed. It dynamically stabilizes through interaction itself for some time. This thematic interest highlights particular micro-affordances as more interesting than others.

To recap, the dancers’ dynamic entanglement reshapes individual affordances extremely rapidly. As soon as the knee lock occurs the dancers *co-modulate* (Kimmel & Rogler, 2018) each other’s affordances in real time. This happens by constraining each other’s degrees of freedom, but also by furnishing each other with unexpected constellations, which provide interesting challenges or possibilities to subvert their habits. The physical interplay and the resulting interaction-based emergence impacts the individual affordance fields dramatically. The dancers are negotiating the joint configuration with high mutual responsiveness. In part this happens with continuity, but in the last sub-sequence the male dancer’s entire affordance field abruptly flips when the partner messes with his ongoing action by pulling away. In this way, affordances perceived by each indivdual become mutually reactive.

### Interaction-based emergence, problem generation, and risk management

A striking facet is how unpredictably the joint ecology evolves through the “give and take” between dancers, a highly dynamic flow of affordances. Both dancers are exposed to a continuous stream of emergent, short-lived micro-affordances, which require unhesitating creative responses. Their actions constantly and massively alter their joint milieu. Ever new affordances are generated through the dynamics of interpersonal engagement, with gravity as a third partner of sorts.

Not only do affordances change all the time, the way they change *causally* depends on interaction. They are heavily contingent on what the dance couple did last and which dynamic configuration resulted. They equally depend on how the physical interplay self-organizes in micro-time. The mutual amplifying effects of the physical entanglement in the later stages of our example dramatically illustrate this. Small movements of one person can have a huge impact on the other person’s situation and affordances. As complexity theorists would say, the dynamic is poised “at the edge of chaos” and far from predictability (Hristovski et al., 2010; 2011). Non-linear effects often emerge from simultaneous initiatives, when each person modulates what the other does, or when one person messes with the joint dynamic. In these dynamic interpersonal negotiations affordances acquire non-linear properties themselves. An affordance can easily flip into its opposite with minimal effort (see Kimmel & Rogler, 2018).^3^ Thus, the situated dynamic is what generates unconventional or surprising affordances. Affordances presuppose the embodied coupling itself, reflecting the unique path-dependent interaction and its (at least immediate) history. The affordance field that presents itself emerges “transactionally” and cannot be attributed to either of the individuals (cf. Kimmel & van Alphen, 2022). Since creative opportunities are caused by the reciprocal engagement a methodologically individualist analysis is inadequate. Instead, it becomes necessary to analyze the *self-organizing causalities of interaction.* These emergent process dynamics can “make or break” specific affordances.

The fact that improvisation is much of the time about leaving the beaten path and, especially, risk taking calls for a broader perspective on affordances than is usual. Behavior need not be strictly adaptive as we have seen in the sub-sequence of the dance where creative effort went into *problem finding*, the counterpart to creative *problem solving* (Runco, 1994). This accentuates the value of introducing constraints and what we might call deliberate “non-affordances.” Somewhat paradoxically, a challenging situation was created on purpose that hardly affords any good immediate actions, but in response to which active affordance-creating or affordance-seeking skills must kick in. The second implication of risks and possible failure is how important capabilities for salvaging dwindling affordances are. Creative fixes and troubleshooting are integral to the improviser’s skill set. Related, in a risky and dynamic environment we see frequent brinkmanship skills, which can be nicely elucidated in an affordance perspective: Brinkmanship can mean making do with not-so-good affordances through inventive action variants and optimizing technique, and in certain cases also switching around such “non-affordances” into something useable (Kimmel & Rogler, 2018).

## Perception, action, and creative orientedness

It is worth pausing to take stock of the multiple ways that affordances feature in our two case-sketches before discussing the theoretical implications.

### How affordances mediate creativity

At times, people “bump into” a creative option. Such on-the-spot affordance “finding,” and similarly perceptual search for unconventional affordances, form recognized creativity mechanisms (e.g., Withagen & van der Kamp, 2018). For example, this happened in our crafts sketch at moments of serendipity and in the dance case when the intersecting legs suggested intensified contact. Here, perceptual ability underlies creative expertise.

We must not over-emphasize this mechanism at the expense of others. It has been relatively neglected that many non-conventional affordances must build on (sometimes extended) active efforts to develop or generate them.^4^ Such *affordance shaping* (Kimmel & Rogler, 2018) consistently runs through both our case-studies. In fact, affordance finding frequently transitions into affordance shaping, for example, when a serendipitous feature of the vase’s silhouette was further accentuated and in the dancers’ knee lock, the “interaction problem” deliberately created by accentuating the prior position through closing the legs fully.

Affordance shaping is fundamental because not all affordances are ready-to-hand. Agents must move or manipulate things to bring them into reach. Accordingly, scholars need to find ways to describe not-yet-so-good options that “beckon” in the environs (Kimmel & Rogler, 2018, p. 210), pointers to proto-states for affordances, as well as directed activities of affordance-enabling or -shaping. Importantly, creative activity frequently builds on conventional affordances to build more interesting ones down the line, for example, by transforming materials. In our crafts example we have seen technical basics that provide stepping stones. Thus, what is at first afforded may be no big creative thing, yet provide an anchor point for bringing more unconventional affordances into being.

Affordances are often *indirectly* generated. Creators may stimulate system reactions “at a remove” to provide creative springboards. In the CI dance, the discussed moment of problem finding through the knee lock deliberately stimulate a new dynamic, without knowing what kinds of affordances would arise from it (i.e., the action was non-deterministic). Creators report inviting interesting affordances by gravitating towards a productive area, use (self-) challenges or “rock the boat”/employ system perturbations. Strategies known as *niche shaping* (Ramstead et al., 2016) may also be used. These may follow relatively unspecific aims, but with some broad class of affordances in mind.

Finally, creativity famously comes to the prepared. Strategies such as preparing the clay in the ceramics example are affordance-enabling. Creators also keep a system productive or “generative,” through technical best practices (workshop maintenance and monitoring the state of the material in crafts or good warm-ups in dance). Such activities are not constitutive of specific affordances, yet they make a set of possible ones likely. Since creators are highly aware of systemic boundary conditions shaping the field of affordances (Bruineberg & Rietveld, 2014) they can actively “cultivate” their ecology. For example, picking a new tool or setting up the action space in specific ways influences what can emerge later.

### Learning from the post-cognitivist field

These empirical sketches point to a continuum of behavioral modalities spanning multiple timescales which contribute to creativity: creative finds, affordances that must be shaped, developed or “turned around”; and enabling preparations so creativity-prone affordances can arise in the first place. Note that only in the first category is perception the main locus of creativity; our analysis notably includes activity as well as a person’s wider orientedness towards the ecology as a site of analysis (see below).

This suggests a holistic perspective on *affordance-mediated creative pursuits*, which treats actions, intentions, and imaginations on a par. We believe that an affordance-focused analysis works best in a broader theoretical context. Notably, distributed creativity, material engagement theory, and enactivist approaches look at affordances with a new twist. Glăveanu’s (2014b, 2020) socio-cultural theory of creativity, for example, underscores that action both engages existing affordances and generates new ones in the context of audiences and artifacts. Similarly, Malafouris and Koukouti (2022) draw on a broad range of theoretical resources to explain how a potter can “engage situational affordances that initially are not perceptible, or are only partially perceptible” (p. 14).

The importance of moving agency center stage is forcefully illustrated by enactivism (see Ward et al., 2017), which envisages a, broadly speaking, “constructive” relationship of agents to milieus and steers clear of blind spots discussed in section 2 (see debates between enactivists and ecological psychologist: Baggs & Chemero, 2018; Flament-Fultot et al., 2016; McGann, 2020). Following Varela et al. (1991), enactivism stresses how the ecology constrains and informs living beings, yet also speaks of these beings as “bringing forth their world.” Enactivists define perception as involving sensorimotor enactment and accordingly emphasize that agents do not simply “respond to” or “resonate with” the ecology (Varela et al., 1991, p. 204). Agents possess considerable autonomy in orchestrating their external interchanges. This emphasis makes room for theorizing creative autonomy, as opposed to a responsiveness-based view.

### Creative orientedness

In “bringing forth the world,” what a creator does, and how, matters. Benefiting from creativity-prone affordances depends on skillful directedness of action. We propose to speak of this factor as *creative orientedness*, which determines whether an unconventional affordance will likely emerge (or be noticed). It depends on micro- and macro-strategies of interaction to cultivate or invite new options, but also on how a context is approached, ways of setting up the ecology, as well as a person’s backdrop of preparedness and habits, how she deports herself. All these are a locus of creative expertise.

Creative serendipity illustrates the importance of the background. Many a “happy accident” becomes possible through prior creative orientedness. Unconventional affordances that unexpectedly pop up may not be blind luck. Chance famously favors the prepared. Similar creative orientedness can be seen in how practitioners balance openness with constraint in their actions (Laamanen & Seitamaa-Hakkarainen, 2014; Petre et al., 2006) and in how they modulate what emerges from the process. In our crafts example the choice of the beautiful but precarious porcelain clay reflected the practitioner’s risk taking in pursuit of the higher aesthetic potential of the material.

To conceptualize how the orientedness of the agent shapes creativity pragmatic approaches to cognition are instructive. Engel describes a mechanism termed “directives” (Engel, 2010), that is, “large-scale dynamic interaction patterns that emerge in a cognitive system” (Engel et al., 2013, p. 206). Directives create dispositional orientations for action, guide a process towards particular possibility spaces, and in turn give rise to affordances. They are ecology-sensitive, yet also open enough to accommodate the fundamental non-determinism creative action possesses. Building on this, Davis et al. (2015) describe creativity as a fundamentally improvisational modality guided by loose directives, which can be “fluidly defined, refined, or discarded altogether” (p. 119). They propose that directives is to select “a filter for perception that […] enables a perception-based reasoning process.” (p. 118). Thus, while a creative agent activate particular dispositions for (e.g., explorative) action, she will typically embrace particular attentional filters. From this perspective, what defines creative experts is to know “how to direct her attention and manipulate the flow of sensory information through interactions with the environment to explore and evaluate possibilities for further action.” (p. 122).

### Partial creative sightedness

We may now take a closer look at how perceptual skills figure in the fluid fleshing out of a creative direction. The underlying puzzle is appositely captured by Christensen as a *search dilemma* (2002, p. 70): How it is possible to search for something you don’t know or does not even exist yet? Creative practitioners evidently cannot know the full outcome before the fact. So of what kind can anticipatory functions be to give their activity a general directionality? Christensen argues as follows: “Searching does not necessarily imply that what we search for is ‘out there’ (in its entirety) prior to the search, just as it does not imply that we have an idea of what we are searching for [....]” (p. 57).

Meanwhile, creativity scholars have reclaimed “partial sightedness” (Dietrich & Haider, 2015) to contrast expert creativity with blind variation and selection. Creators frequently have a prospective feel for the possible. Someone possessing partial sightedness can effectively “zoom in on a probable region of the solution space” (ibid., p. 901). This means that creators frequently think a specific creative pursuit worthwhile even while not knowing the precise future yet, an intuitive foresightedness that emerges from the person's experiential knowledge. The question is how they identify such promising paths. We may assume that “signposts” can be perceptually detected at various junctures in the process itself. A creative outcome is often not proximally afforded; it may however be recognized as tentative horizon.

In other words, creators are often capable of detecting creative “kernels” worth cultivating (Kimmel & Hristova, 2021). They recognize information that not yet specifies a fully creative actionable, but points the way. Subtle affordance-like pointers may specify such creative “kernels,” which help distinguish afforded from non-afforded next steps and decide how to create “springboards” for creative development. This would require a kind of proto-affordance that indicates promise, although not certainty. The identification of such “kernels” allows creators to invest into some good direction to pursue.

To summarize, we hypothesize that signpost-like proto-affordance allow detecting conditional potentialities in constellations not affording anything in and of themselves yet, but if developed and acted upon they may become potent. Studying this phenomenon further seems an important future direction for scholarship to respond to the discussed problem of the “not-yet-real.”

## How do affordances relate to imaginative and generative functions?

Further advancing the debate requires discussing the scope of affordances in relation to mechanisms such inspiration, imagination and mental recombination. How are the latter made convergent with material or interpersonal solicitations.

### How affordances and intentions converge

The empirical observation to start from is that creators regularly report how personal preferences, themes, or sources of inspiration work “underneath” the engagement with the ecology. An ecological paradigm must not lose out of sight such bona fide “mental” capacities. Even if we recognize perception and action as explanatory loci of creativity we should not espouse a behaviorist fallacy of “creativity in the world” purged of the mental. As Glăveanu (2020, p. 348) puts it, a “sociocultural theory is not opposed to individual-level or cognitive theories of creativity.” We must therefore seek an explanation of how mechanisms such as the heuristics, inspirations, imaginative or combinatoric capacities get “plugged into” the creative engagement with affordances.

Creative aims and interests in part emerge as *intentionality-in-action*, by engaging with affordances, in the interaction. But another part may belong into the category of intentionality-before-action, i.e. creative interests that transcend the situation (even when remaining open to guidance from it).^5^ For example, dance improvisers may introduce inspiring observations made in the public sphere or personal pre-occupations as creative themes (Kimmel et al., 2018). They integrate these into interaction when a compatible moment arises. This is quite natural because intentions of dancers have nested levels, from general and only broadly constraining slow dynamics of intention to fast dynamics that flesh the former out as action is generated. That is, broad creative intentions (quite unlike plans) may precede the moment, but are then specified further with respect to the present field of affordances. Which of the many affordances are selected, however, typically follows the broader intention.

The imagination is a major medium of creative intentions. The imagination, in some respects, co-evolves with the affordance field, given its dynamical and enrichment-prone nature (Rucińska, 2021). After Koukouti and Malafouris (2020, p. 30), the imagination is “entangled with matter and the affordances of things.” It is anchored in the perceived physicality of the situation, such that in a *material imagination* the actual and the possible can be co-experienced. The phenomenological relevance is shown in our crafts example.

This notwithstanding, any one-sided emphasis on the imagination’s situated anchoring neglects its situation-transcending features. Able creators may look beyond the manifest ecology by bringing to bear extraneous inspirations, analogies, divergent thinking, and other modes that disengage from situated givens. Davis et al. (2015) quite rightly build their enactive model around the assumption that creativity can “unclamp perception” and hereby temporarily disengage from a task. If we take this possibility seriously the creative imagination cannot be a mere extension of a given situation; we cannot fully “immanentize” it.

### Subsumption or partnering?

We may contrast two ways of connecting the imagination with affordances. van Dijk and Rietveld (2020) recast the imagination as fed by perceived affordances, a type of global affordance perception. They depict prospective functions as “opening up to larger-scale affordances” of a creative pursuit, given that “what is being imagined is constitutively tied to the history of activity and the possibilities for future activity available” (p. 2). We agree that creative anticipations need to be ecological constrained. Yet apart from over-exerting the affordance concept itself, this position risks trivializing the imagination. Imagining is phenomenologically richer than just a sophisticated kind of perceiving ecological potentials. Directly defining the imagination in terms of affordances risks immanentizing it and hereby losing out of sight its situation-transcending aspects.

We therefore prefer to define the imagination as a source of creativity in its own right, not just an extension of affordances. This means giving affordances partner concepts. Glăveanu’s *affordance-perspective theory* (2020) does this. It posits a dialect between affordances and wider perspectives that impart creative orientation (and can be progressively enriched, adapted, or reoriented while interacting). In our view, perspectives are a helpful concept to elucidate why and how creators constrain the engagement, set up the ecology, and hereby highlight particular affordances as relevant. This confers a non-arbitrary directedness, which nonetheless stays suitably open to novelty, surprise and chance.

For instance, identifying affordances as serendipitous requires the “perspectival” wisdom to recognize their utility (Ross & Vallée-Tourangeau, 2021b). Perceiving alone is insufficient; a perspective is needed as a yardstick. Perspectival thinking looms large in our crafts example, which allowed a set of spatio-temporally distributed affordances to be developed into a coherent aesthetics. Perspectival thinking also underlies classics of creativity research: re-framing insight problems to overcome “functional fixedness” (Duncker, 1945; Wertheimer, 1945). The solution involves detecting a non-canonical affordance, which involves a double operation of perceptual noticing (“seeing as”) and re-interpreting the task itself. The problem solver adopts novel ways of perceiving while modifying the “psychological” stance to a problem context.

### The mental life of affordances

Another recent idea is to expand the meaning of “affordance” itself. What if we stopped thinking of affordances as being exclusively for bodily actions, for example? McClelland (2020) introduces “*mental affordances*”, hypothesizing that these are “opportunities to perform a mental action” (p. 401). This idea is useful, because affordances can indeed trigger creativity related thought processes, for example in crafts, about the right tools to realize something new or the best strategy to use when time runs out.

Furthermore, combinatorial generativity, a central topic of creativity scholarship, can be brought into the fold of affordance theory as well. Novelty that arises through recombination, for example, capitalizes on the “mental virtuosity” (Stachó, 2018) of expert improvisers. Glenberg and Robertson (2000), in studies of action language, analyze how movement primitives or known object properties can be combined to create new affordances (cf. Glenberg, 1997). They claim that this involves mental pre-simulation of *affordance mesh* whereby novel functionalities are envisioned in light of a desired action goal. One basic scenario Glenberg and Robertson discuss involves non-canonical affordances (2000, p. 385): Most people can “see” that one can dry one’s feet with a shirt after swimming because the object affords this, although this is not the typical use. Beyond this, non-existent affordances can be literally created by combining multiple mundane affordances, such as when a camper figures out that a sweater affords filling with leaves to make a pillow. By imagining how different actions or objects “synergize” genuinely creative possibilities emerge. We also saw this affordance mesh in the example of the ceramicist who imagined how an already given feature of a vase could be complemented through non-yet-existing ones. When someone meshes affordances they imagine feature combinations in view of an ends. Novel configurations are emulated via “virtual” affordances. Such acts of the creative imagination are grounded in embodied experience, yet also transcend the momentary situation.

Finally, it seems tempting to integrate generativity into an ecological perspective via Hutchins’ (2005) suggestion that “material anchors” license productive conceptual activity. He notably includes in this the guidance one can receive from “imagining the manipulation of physical structure” (p. 1575) and imaginative schemata that become co-projected into a blend with material cues.

Overall, how the material, the imagined, and the conceptual converge in creativity is the next academic frontier, which calls for bridge-building between interactionist and mentalist species of scholarship, and a non-dogmatic stance vis-à-vis representational posits such as “embodied emulation” or “conceptual blending”. The reward for an ecumenical stance is leverage on the complex interplay of affordances with other facets of creativity.

## Conclusion

Materiality, embodiment, and interaction provide vital resources for creativity, as has been stressed by Schön, Sawyer, Malafouris, Valée-Tourangeau, Ross, Torrents, or Hristovski, amongst others. Affordance theory offers a good analytic prism to develop this ecological-interactive and not mentally encapsulated perspective. The creative role of affordances is to specify action opportunities that are themselves creative or indirectly contribute to “useful novelty.” But how can we articulate this perspective for it to gain traction with creativity scholars? Although recent work by scholars like Glăveanu or Rietveld’s group is suggestive the task is non-trivial. Several epistemological and methodological facets await discussion. We have presently tried to contribute to this task, while trying get the measure of the scope of affordances for creativity scholarship.

Gibsonian thought, with its emphasis on the resourcefulness of the ecology, can help explain part of the creativity puzzle, with respect to “how something can come from nothing” (Christensen, 2002). However, its disadvantage is that it leaves little room for subjective, constructive process and mental generativity mechanisms. Therefore, a middle position between realist and constructivist concerns will provide a likely path forward; a continued dialogue with other “4E” cognition branches will prove essential here.

Our micro-ethnographic case-sketches indicate that affordances contribute to creativity in virtue of more than a perceptual ability. “Bumping” into or deliberate perceptual search for affordances is just one aspect (and even a serendipity is more than mere affordance finding). Much creativity rests on creating and transforming mundane affordances or actively shaping the ecology. Creators may probabilistically “invite” creative resource or provide enabling stepping stones, and, through their wider intentional and embodied orientedness towards the milieu, influence which kinds of affordance will manifest.

How should we talk about ecological and interactive functions of creativity then? We have shown that moments that afford immediate creative action exist, but in others “the creative” is only the attribute of an extended practice. This suggests starting the analysis from *affordance-mediated creative activity* (Kimmel & Groth, 2023) rather than narrowly conceived “creative affordances.” This activity is always relative to multi-timescale engagement with the ecology, from which emergent system dynamics arise.

Furthermore, saying the creative agents are responsive to or seek resonance with their ecology is insufficient, as it veils key characteristics of experts: their creative autonomy and “vision,” their directedness of interest, their niche shaping and tool making, and their ability to impose selective constraints. Accordingly, an ecological-interactive paradigm must discuss intentions, imaginations of the “not-yet-real,” and mental combinatorics. These mechanisms operate within certain ecological constraints, yet also reach beyond the givens, and how they relate to affordances requires further clarification. In continuing this debate we invite readers to think about how to best acknowledge the fundamental parity of agent and ecology as complementary, but also confluent loci of the creative.

## Notes

1. This would include the *geneplore* model (Finke et al., 1992; Ward et al., 1999) and studies on how goals co-evolve with means (Dorst & Cross, 2001). Even studies of material creativity media such as Mace and Ward (2002) emphasize the exploration of ideas, not material fiddling or manipulation.

2. For example, learning is seen as increasing (perceptual) *attunement* to the environment making affordances accessible (E. J. Gibson, 1988). Note also that such relationality claims originate in an aggregate perspective on species and their environment based on the idea that a species is attuned to their niche. A similarly aggregate focus has in recent times been transposed to “forms of life,” including social groups (Rietveld & Kiverstein, 2014) and socio-cultural fields (Heft, 2007).

3. The fact that the CI system lacks a designated leader or follower role makes it especially prone to non-linear dynamics (as does the avoidance of chaining pre-defined “moves”).

4. When Gaver (1991) coined the idea of *sequential affordances* he used this to refer to step-wise discovery: New affordances reveal themselves when a prior affordance is acted on. However, sequential affordances can also involve enacting directed steps in the hope some specific affordance comes aboutr.
